# Intracranial pressure monitoring in intensive care: clinical advantages of a computerized system over manual recording

**DOI:** 10.1186/cc5155

**Published:** 2007-01-18

**Authors:** Elisa Roncati Zanier, Fabrizio Ortolano, Laura Ghisoni, Angelo Colombo, Sabina Losappio, Nino Stocchetti

**Affiliations:** 1 Neurosurgical Intensive Care Unit, Department of Anesthesia and Critical Care Medicine, Milan University, Ospedale Maggiore Policlinico, Mangiagalli, e Regina Elena, Fondazione IRCCS, Via Sforza n 35, 20122 Milano, Italy

## Abstract

**Introduction:**

The presence of intracranial hypertension (HICP) after traumatic brain injury (TBI) affects patient outcome. Intracranial pressure (ICP) data from electronic monitoring equipment are usually calculated and recorded hourly in the clinical chart by trained nurses. Little is known, however, about how precisely this method reflects the real patterns of ICP after severe TBI. In this study, we compared hourly manual recording with a validated and continuous computerized reference standard.

**Methods:**

Thirty randomly selected patients with severe TBI and HICP admitted to the neuroscience intensive care unit (Policlinico University Hospital, Milan, Italy) were retrospectively studied. A 24-hour interval with ICP monitoring was randomly selected for each patient. The manually recorded data available for analysis covered 672 hours corresponding to 36,492 digital data points. The two methods were evaluated using the correlation coefficient and the Bland and Altman method. We used the proportion test to analyze differences in the number of episodes of HICP (ICP > 20 mm Hg) detected with the two methods and the paired *t *test to analyze differences in the percentage of time of HICP.

**Results:**

There was good agreement between the digitally collected ICP and the manual recordings of the end-hour values. Bland and Altman analysis confirmed a mean difference between the two methods of 0.05 mm Hg (standard deviation 3.66); 96% of data were within the limits of agreement (+7.37 and -7.28). The average percentages of time of ICP greater than 20 mm Hg were 39% calculated from the digital measurements and 34% from the manual observations. From the continuous digital recording, we identified 351 episodes of ICP greater than 20 mm Hg lasting at least five minutes and 287 similar episodes lasting at least ten minutes. Conversely, end-hour ICP of greater than 20 mm Hg was observed in only 204 cases using manual recording methods.

**Conclusion:**

Although manually recorded end-hour ICP accurately reflected the computerized end-hour and mean hour values, the important omission of a number of episodes of high ICP, some of long duration, results in a clinical picture that is not accurate or informative of the true pattern of unstable ICP in patients with TBI.

## Introduction

The presence of intracranial hypertension or high intracranial pressure (HICP) (> 20 mm Hg) after traumatic brain injury (TBI) affects patient outcome [1] and calls for prompt recognition and treatment. Accurate monitoring of intracranial pressure (ICP) is therefore essential in neuro-intensive care, and the utility of different ICP sensors has been explored extensively [2]. In most ICU settings, clinical data from the monitoring equipment are usually summarized hourly in the clinical chart by trained nursing staff. Early studies of pharmacological treatment for TBI [3] reported ICP as entered by the investigators at every end-hour interval, and this policy has been used in a variety of subsequent pharmacological trials [4,5]. However, whether this method reflects the real patterns of ICP in these acute cases involving unstable HICP is poorly understood [6] and whether manually recorded end-hour values are representative of the real pattern of ICP remains unclear.

In our unit, we have been using a comprehensive computerized system specifically designed for ICP analysis, coupled with traditional manual recording by the nurses, for many years. This five year retrospective study sought to compare the accuracy and clinical fidelity of manual hourly ICP recordings with the computerized data to verify the capability of the two systems to properly capture ICP increases and to adequately rank the severity of ICP in single TBI cases.

## Materials and methods

Among 293 TBI patients, admitted to the neuroscience intensive care unit (ICU) at the Ospedale Maggiore Policlinico (Milan, Italy) from 1 January 1997 to 1 December 2002, electronic recordings available for 170 of those patients were examined. The inclusion criteria for this study were age of more than 14 years, severe TBI (post-stabilization Glasgow Coma Scale [GCS] of less than or equal to 8), ICP and cerebral perfusion pressure (CPP) monitoring for at least two days, and ICP higher than 20 mm Hg for at least 25% of the monitoring time. Sixty-two cases fulfilled these criteria. These 62 cases were coded for anonymity and patients were listed in chronological order. For this retrospective study, one author (FO) randomly chose 30 cases from the list and for each patient, randomly selected one 24-hour interval of all ICU days with ICP monitoring documented in the medical chart.

In our unit, trained nurses manually enter the end-hour ICP value every hour on a form specifically designed for recording physiological measurements. Concurrently with the manual recording, a computerized system continuously acquires ICP and CPP. Briefly, data from the ICP monitoring system are continuously sent to a Macintosh computer (Apple Computer, Inc., Cupertino, CA, USA) through an analog-digital converter (MacLab; ADInstruments Pty Ltd., Castle Hill, Australia). Therefore, it is possible at any time to write a clinical note on the computerized chart that is stored together with the ICP data. The computer records 10 points per minute, so more than 430,000 points were available for this analysis. To exclude potentially inaccurate data (such as interruption of ICP readings due to transducer zeroing, cerebral spinal fluid sampling, and so on), all traces were visually reviewed and all artifactual data were removed. This procedure discarded 9.5% of data points.

The manually recorded data available for analysis covered 672 hours. In 48 instances, patients were moved for computed tomography scans and so on; accordingly, the digital data for these 48 missing hours were excluded from analysis, leaving 36,492 data points that were averaged per minute and per hour.

To address the capability of the manual system to properly capture ICP increases and to adequately rank the severity of ICP, comparisons with the digital tracing were made and the digital tracing was analyzed in five ways: (a) The end-hour minute ICP was identified. (b) The average ICP for each hour was calculated. (c) Episodes of HICP (> 20 mm Hg) were identified and their durations were calculated. (d) The number of five minute electronic HICP episodes per hour was calculated and each hour was assigned to one of three different categories: (i) no HICP episodes, (ii) one to five HICP episodes, or (iii) ICP constantly higher than 20 mm Hg. (e) The percentage of time with an ICP of greater than 20 mm Hg was also calculated by filtering the digital data using proprietary software (Super ICP analyzer, author AC).

The two methods were evaluated using the correlation coefficient and the Bland and Altman method [7]. Because the electronic system was considered the reference value, it was entered on the abscissa of the Bland and Altman plot [8]. We used the proportion test to analyze differences in the number of episodes of HICP detected with the two methods and the paired *t *test to analyze differences in the percentage of time of HICP.

The hospital ethics committee granted permission for prospective collection, storage, and research analysis of clinical and electronic data. Each patient's next of kin was informed, and written consent was obtained with the understanding that routine clinical and monitoring data were to be collected, stored in a database, analyzed for research purposes, and possibly published (once rendered anonymous).

## Results

### General data

Ten women and 20 men ranging in age from 16 to 54 years (average 29 ± 12 years) were included in the present analysis. The post-stabilization GCS was calculated for each patient and the median was determined to be 5, whereas average ICP was 20.3 ± 2.9 mm Hg and average CPP was 68 ± 5.7 mm Hg. For each patient, ICP was monitored for 6 ± 3 days (with a range of 2 to 10 days). The ICP catheters were placed in the subdural space in 24 cases, in the ventricles in four cases, and in the parenchyma in two cases.

### End-hour ICP

**Figure 1:**
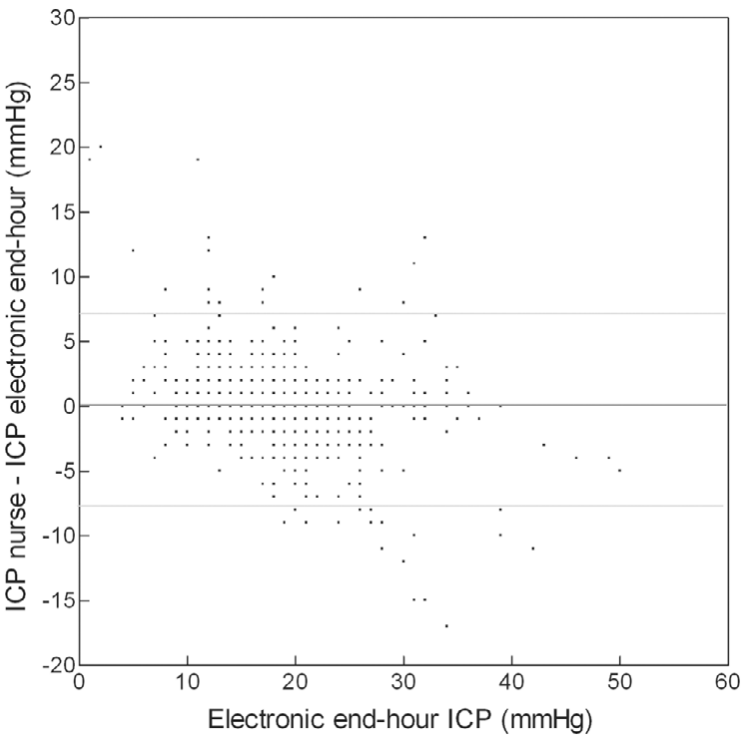
Bland and Altman's graph for end-hour intracranial pressure (ICP). The dark line shows the mean difference between manual and digital recording methods (0.05 mm Hg). The two light lines show the limits of agreement (-7.28 and +7.37).

The ICP entered on the clinical chart was compared with the corresponding digital recording (average of the last minute for every hour). The linear correlation between the two techniques was very good (*r *= 0.89) (Figure 1). The mean difference between the two methods was 0.05 mm Hg (standard deviation [SD] 3.66), and 93% of data were within the limits of agreement (+7.37 and -7.28).

### Mean hour ICP

**Figure 2:**
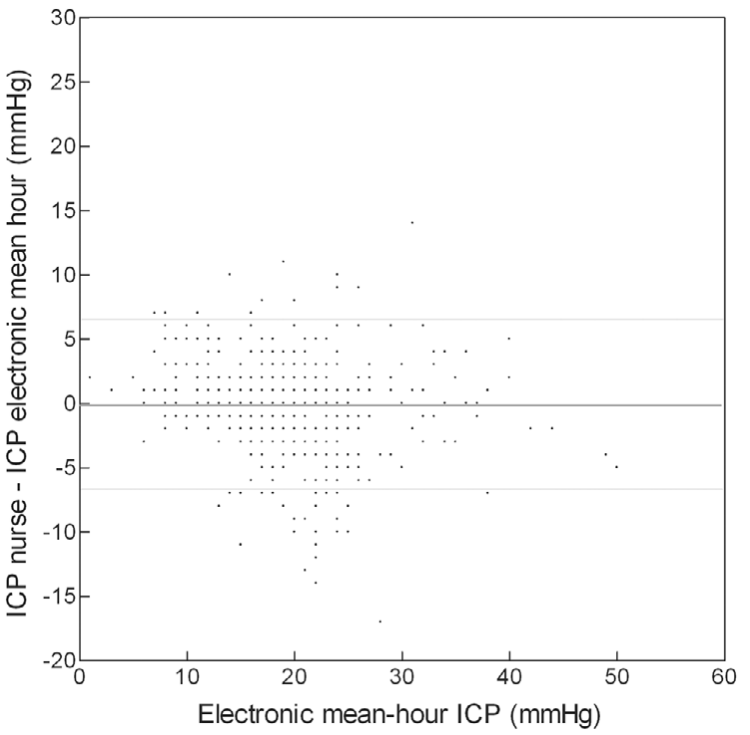
Bland and Altman's graph for mean hourly intracranial pressure (ICP). The dark line shows the mean difference between manual and digital recording methods (-0.08 mm Hg). The two light lines show the limits of agreement (-6.84 and +6.68).

The electronic average for every hourly digital measurement was compared with the corresponding end-hour value manually recorded by the nurses. Again, the correlation was good (*r *= 0.85) (Figure 2). The mean difference between the two methods was -0.08 mm Hg (SD 3.38), and 93% of data were within the limits of agreement (+6.68 and -6.84).

### Episodes of HICP

From the continuous digital recording, we identified 351 episodes of ICP greater than 20 mm Hg lasting at least five minutes and 287 episodes lasting at least ten minutes. However, end-hour ICP greater than 20 mm Hg was manually recorded for only 204 episodes. The proportion of missed data was therefore 42% (95% confidence interval [CI] 36% to 46%) for episodes longer than five minutes and 29% (95% CI 29% to 39%) for longer-lasting episodes. The proportion test indicated a significant difference (*p *< 0.0001) between the numbers of ICP increases identified by the digital and the manual systems.

### Numbers of five minute electronic HICP episodes per hour

**Figure 3:**
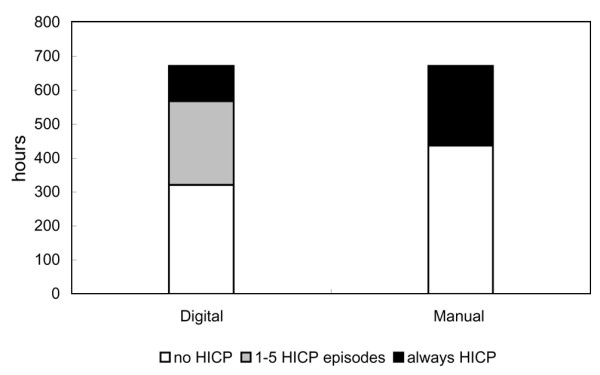
Bar graphs depicting the capabilities of the digital and manual systems to capture increases in intracranial pressure (ICP). The bar on the left represents the number of hours identified with the digital system; an ICP always greater than 20 mm Hg (continuous intracranial hypertension [HICP]) is indicated in black, one to five episodes of ICP greater than 20 mm Hg in gray, and no HICP (ICP > 20 mm Hg) in white. The bar on the right represents the number of hours identified with the manual system; an ICP greater than 20 mm Hg is indicated in black and an ICP less than 20 mm Hg in white.

Over the 672 hours, using the digital recording system, we identified 321 hours with no documentable episodes of HICP, 247 hours containing one to five HICP episodes, and 104 hours with a continuous HICP. Conversely, based on the manual system, 437 hours of no HICP (< 20 mm Hg) were reported and only 235 hours of HICP were detected and recorded (Figure 3). Therefore, 116 hours with at least one episode of HICP documented by the digital recording system were labeled 'benign' using the manual recording system.

### Percentage of time of HICP

The percentage of time of ICP greater than 20 mm Hg was calculated (with proprietary software) from the digital data as follows:

Number of minutes of ICP greater than 20 mm Hg × 100/total minutes.

**Figure 4:**
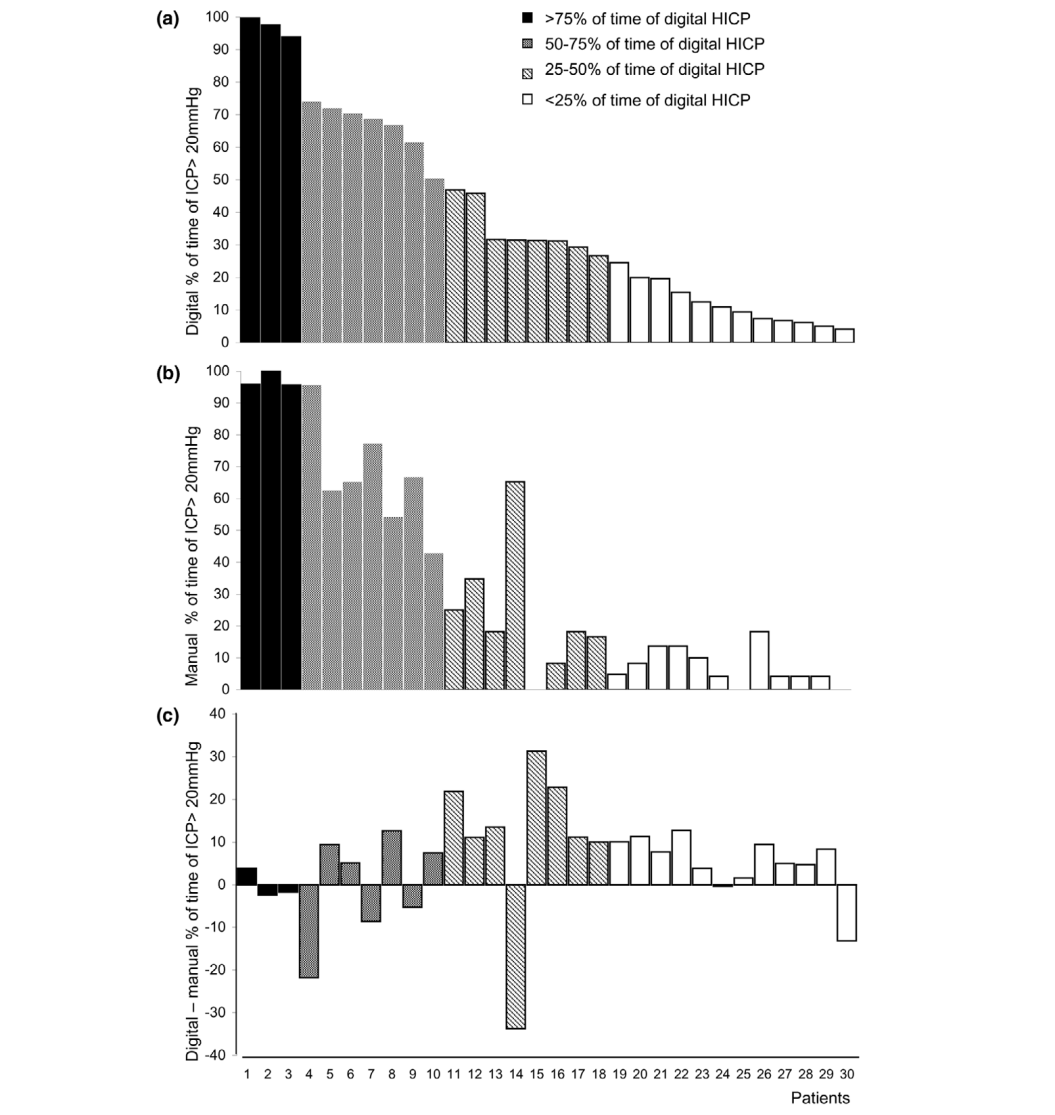
Bar graphs depicting the **(a) **digital and **(b) **manual percentages of time of intracranial hypertension (HICP) (> 20 mm Hg) for each patient and **(c) **the difference of digital and manual percentages of time of HICP. Based on the digital results, patients are arranged in descending order and are divided in quartiles. The upper quartile (black bars) consisted of patients with a percentage of time of digital HICP of more than 75%, the third quartile (gray bars) consisted of patients with a percentage of time of digital HICP of 50% to 75%, the second quartile (hatched bars) consisted of patients with a percentage of time of digital HICP of 25% to 50%, and the lower quartile (white bars) consisted of patients with a percentage of time of digital HICP of less than 25%. HICP.

Overall, the digital percentage of time of ICP greater than 20 mm Hg was 39%. The digital percentage of time of ICP greater than 20 mm Hg for each patient is shown in Figure 4a and was more than 75% in three patients (upper quartile, black bars), between 50% and 75% in seven patients (gray bars), between 25% and 50% in eight patients (hatched bars), and less than 25% in 12 patients (lower quartile, white bars).

The percentage of hours of ICP greater than 20 mm Hg identified with the manual system was calculated as follows:

Number of hours of ICP greater than 20 mm Hg × 100/total hours.

Overall, the manual percentage of hours of ICP greater than 20 mm Hg was 34%. Comparisons between the digital and manual percentages of time spent in which ICP exceeded 20 mm Hg showed that, overall, the manual system dramatically underestimated ICP severity (*p *< 0.05 paired *t *test; Figure 4b).

Furthermore, for each patient studied, the difference between digitally recorded and manually recorded percentages of time in which ICP was greater than 20 mm Hg was calculated (Figure 4c). For example, patient 1 had a digitally recorded percentage of time in which ICP was greater than 20 mm Hg of 99.7% and a manually recorded percentage of time in which ICP was greater than 20 mm Hg of 95.8%, a difference of 3.9%. The agreement between the two methods was fairly good for patients in the upper (black bars) and lower (white bars) quartiles, with a mean difference between the two methods of 6.6% ± 5.6% and 2.7% ± 0.6%, respectively. Conversely, the mean difference between the two methods was 19.5% ± 9.4% for the group of patients in the second quartile (gray bars) and 10% ± 5.8% for the group of patients in the third quartile (hatched bars). These differences in the data highlight how patients with a fluctuating ICP are the most prone to an erroneous detection of ICP severity when only the manual recording technique is used.

## Discussion

The results of the present study demonstrate that there was good agreement between ICP data collected using continuous digital computerized techniques and those data collected using traditional manual recording as long as the end-hour pressure data were analyzed. The limits of agreement were narrow, and the end-hour data entered from the monitor and recorded by the computer were markedly similar. The correlation was also good between the mean hourly values and the end-hour data, even if the 95% limit of agreement of ± 7 mm Hg in borderline ICP cases could be clinically relevant. Moreover, if we had relied only on the manually obtained values, more than one third of HICP episodes would have been missed and not documented. Because the same patients were recorded with both systems, it is impossible to detect any difference in treatment, or in outcome, associated with different methods of ICP data collection. It is very likely that, at the bedside, prompt reaction to ICP increase was the rule, irrespective of the recording method used; what is different is the sum of the ICP data, and we believe that these findings have important implications for ICP-based clinical studies. Because manually recorded end-hour values correlate well with digital recordings, investigators could use these data to obtain a reliable description of ICP over time.

Several papers were published in the 1970s to outline the importance of continuous monitoring for neurosurgical patients [9-11]; however, the differences among recording methods (manual versus digital) are probably expected but rarely considered when ICP is analyzed in the literature.

In the data collected internationally by the Traumatic Coma Data Bank (TCDB), ICP recordings were entered [12] using end-hour values because previous work had shown that 84% of the measurements recorded by nurses were within 6 mm Hg of the electronic recording [13]. On the basis of these same conclusions, the TCDB investigators decided not to enter subjective estimates of hourly HICP and to use end-hour values as a robust physiological descriptor. In a neonatal ICU [14], physiological data (not ICP) stored by computer every second were compared with the single hourly values noted by the nurses. Manual and computer observations showed some significant differences, but they were determined not to be clinically important.

A recent study conducted on 16 patients with severe TBI [6] compared a manual recording system of ICP data with a computerized reference that collected only four data points per hour. In that study, a strong correlation for ICP between the hourly mean values calculated from the 15-minute measurements and the end-hour value as recorded by the nurse (*r*^2 ^= 0.95) was found, and perhaps more importantly, the frequency of perturbation in ICP detected by the 15-minute values was no different from that detected by the end-hour values. The authors concluded that the end-hour ICP was as accurate as more frequent measurements during the hour [6].

Our data, while confirming the excellent and important correlation between end-hour data, bring to light specific and significant differences in the capabilities of the two systems to adequately assess the severity of ICP in individual TBI cases, in which ICP can fluctuate widely. There was a significant difference between the number of individual ICP elevations as identified by the digital versus manual systems, and the average percentages of time of HICP calculated from the two methods also differed significantly. A possible explanation for this which differs from the one previously proposed [6] is that the data used for the comparison in the aforementioned study were limited to end-hour measurements compared with four digital points per hour, whereas our study used 600 data points for the same interval.

Our findings confirm that although the traditional manual system of ICP measurement in the ICU provides an accurate picture, a continuous, computerized digital system stores the whole 'movie,' containing details that may be clinically important but that are not visible in a single 'snapshot.' However, if the whole 'movie' is summarized as the average whole-day ICP, the resulting mean is not far from the one obtained by individual 'snapshots' [15]. Moreover, there are specific limitations concerning the continuous digital recording system, including the necessity to review and edit the data [14]. This improves the reliability of the data but is time-consuming and requires human intervention and judgment, thereby introducing the possibility of human error.

## Conclusion

The end-hour ICP manually recorded by experienced nurses is reliable and provides a robust description of the general ICP trend, but on the basis of this measurement alone, a number of episodes of HICP (some of long duration) may be missed, with the risk of underestimating the severity of a patient's injury and the intensity of treatment required. Depending on the purpose of the data collection, each data system (manual or digital) can better fit the aim. When a detailed analysis of ICP of individual cases is desirable, a digital system with proper filtering appears to be more accurate.

## Key messages

• After severe TBI, the end-hour ICP manually recorded is reliable in describing the general ICP trend.

• To properly capture ICP increases and to adequately rank the severity of HICP, the computerized ICP monitoring shows clinical advantages over manual recording.

## Abbreviations

CI = confidence interval; CPP = cerebral perfusion pressure; GCS = Glasgow Coma Scale; HICP = intracranial hypertension; ICP = intracranial pressure; ICU = intensive care unit; SD = standard deviation; TBI = traumatic brain injury; TCDB = Traumatic Coma Data Bank.

## Competing interests

The authors declare that they have no competing interests.

## Authors' contributions

ERZ participated in the conception and design of the study and drafted the manuscript. FO made substantial contributions to the acquisition, analysis, and interpretation of data and helped to draft the manuscript. LG and SL made substantial contributions to the acquisition, analysis, and interpretation of data. AC participated in the design of the study and performed the statistical analysis. NS conceived of the study, participated in its design, and critically revised the manuscript. All authors read and approved the final manuscript.
